# Black‐Blood Contrast in Cardiovascular MRI

**DOI:** 10.1002/jmri.27399

**Published:** 2020-10-19

**Authors:** Markus Henningsson, Shaihan Malik, Rene Botnar, Daniel Castellanos, Tarique Hussain, Tim Leiner

**Affiliations:** ^1^ Division of Cardiovascular Medicine, Department of Medical and Health Sciences Linköping University Linköping Sweden; ^2^ Center for Medical Image Science and Visualization (CMIV) Linköping University Linköping Sweden; ^3^ School of Biomedical Engineering and Imaging Sciences King's College London London UK; ^4^ Division of Pediatric Cardiology, Department of Pediatrics University of Texas Southwestern Medical Center Dallas Texas USA; ^5^ Division of Pediatric Radiology, Department of Radiology University of Texas Southwestern Medical Center Dallas Texas USA; ^6^ Department of Radiology Utrecht University Medical Center Utrecht The Netherlands

**Keywords:** black‐blood contrast, vessel wall imaging, blood signal suppression

## Abstract

MRI is a versatile technique that offers many different options for tissue contrast, including suppressing the blood signal, so‐called black‐blood contrast. This contrast mechanism is extremely useful to visualize the vessel wall with high conspicuity or for characterization of tissue adjacent to the blood pool. In this review we cover the physics of black‐blood contrast and different techniques to achieve blood suppression, from methods intrinsic to the imaging readout to magnetization preparation pulses that can be combined with arbitrary readouts, including flow‐dependent and flow‐independent techniques. We emphasize the technical challenges of black‐blood contrast that can depend on flow and motion conditions, additional contrast weighting mechanisms (T_1_, T_2_, etc.), magnetic properties of the tissue, and spatial coverage. Finally, we describe specific implementations of black‐blood contrast for different vascular beds.

**Level of Evidence:**

5

**Technical Efficacy Stage:**

5

MAGNETIC RESONANCE IMAGING (MRI) allows high‐resolution tomographic imaging with excellent soft tissue contrast. The MRI contrast mechanism can be related to magnetic properties (eg, longitudinal relaxation time—T_1_, and transverse relaxation time—T_2_) or physiological properties (eg, microscopic water diffusion, capillary perfusion, or macroscopic blood flow) and depends on the specific pulse sequence. In many vascular applications, for example, to assess plaque burden in atherosclerosis^1^ or to detect deep vein thrombosis (DVT),^2^ it is desirable to suppress the blood signal in order to depict the surrounding vessel wall with high conspicuity using so‐called black‐blood contrast. Black‐blood contrast can also benefit tissue characterization of the myocardium by minimizing the often‐confounding blood signal.^3^

In general, black‐blood contrast can be combined and complement the previously mentioned MRI contrast mechanisms. However, unlike these common contrast types that are ubiquitous in most MRI textbooks, black‐blood contrast has received relatively little attention. In this review we will cover the physical principles of how black‐blood contrast can be achieved by exploiting blood flow or differences in tissue properties (primarily T_1_ or T_2_). Although there are many flavors of black‐blood techniques, depending on the anatomy and additional contrast weightings, there are some common challenges that should be highlighted initially: 1) the tissues of interest, which we will refer to henceforth as static tissue (unless otherwise specified), such as vessel wall and myocardium have magnetic properties (T_1_ ~ 1000 msec and T_2_ ~ 50 msec on clinical field strengths) that complicate the efficient acquisition of high signal‐to‐noise ratio (SNR) images. Even if blood is adequately suppressed, the short T_2_ of the static tissue results in a limited time for data collection before the signal decays, while the relatively long T_1_ imposes SNR penalties on short repetition times. 2) Homogeneous, robust suppression of blood signal is challenging due to a combination of factors (including complex and unpredictable blood flow and its long T_2_), which will be covered in more detail in the following sections. However, initially we will just make the cautionary note that it may not be possible or even desirable to suppress all blood signal and the amount of suppression has to be traded off for SNR, contrast‐to‐noise ratio of the static tissue, spatial coverage, motion artifacts, and scan time.

Despite these challenges, there is a growing interest and increased clinical uptake for black‐blood techniques. In this review we will describe specific implementations of black‐blood MRI for different anatomical areas and in combination with different contrast mechanisms. We will also highlight limitations of current techniques and common artifacts that are particular to black‐blood techniques.

## Physics of Black‐Blood Contrast

### Spin Echo Techniques

One of the most effective methods to suppress blood signal in MRI is to leverage the motion sensitivity of this modality to minimize signal from flowing blood. The spin echo pulse sequence is particularly motion‐sensitive and is therefore well‐suited to yield intrinsic black‐blood contrast.^4^ There are in fact two separate and additive flow‐related mechanisms that contribute to blood suppression for the spin echo pulse sequence; one related to through‐plane flow and the combination of excitation and refocusing pulses the flowing blood experiences, and a second mechanism related to the flow‐related dephasing of transverse magnetization caused by the heterogeneous accumulation of phase for flowing spins in a voxel, primarily in the in‐plane direction. We refer to these mechanisms here as *through‐plane flow suppression* and *motion‐induced intravoxel dephasing*.

#### THROUGH‐PLANE FLOW SUPPRESSION

**FIGURE 1:**
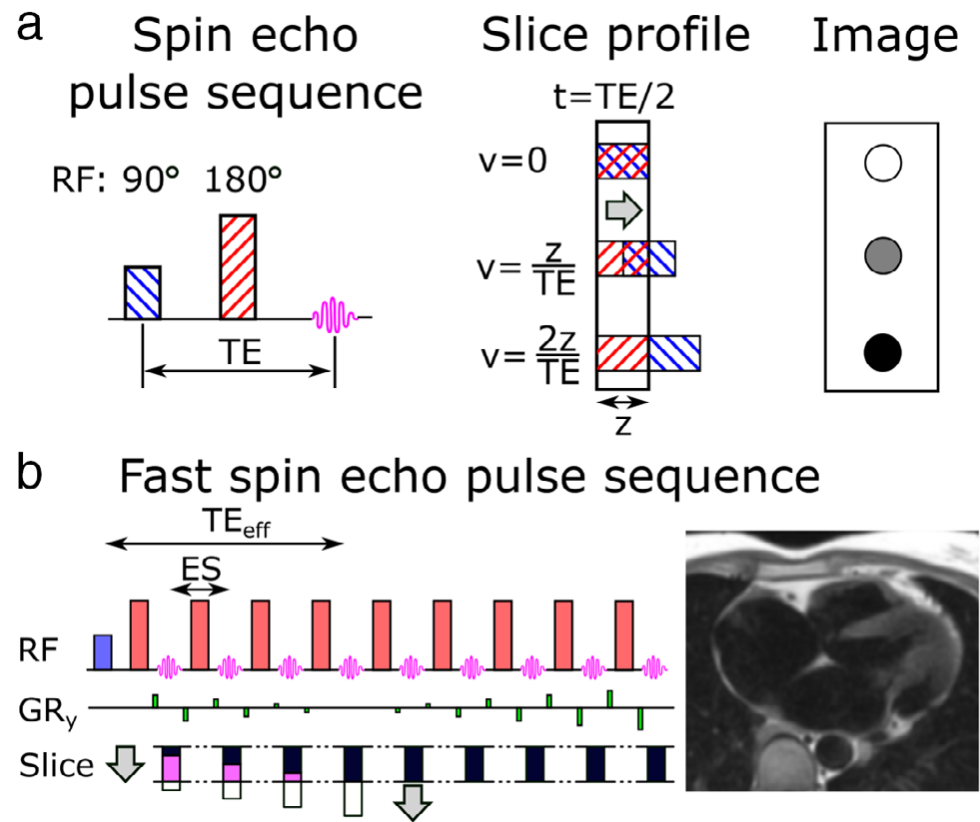
Black‐blood contrast can be achieved with the spin echo technique due to through‐plane flow between the slice‐selective 90° excitation and 180° refocusing pulses (**a**). The slice profile shows through‐plane flow for three velocities (v) at the time t = TE/2: v = 0, v = z/TE, and v = 2z/TE, where z is the slice thickness and TE the echo time. In the first case, the tissue experience both 90° and 180° pulses, yielding the maximum signal at the echo time. In the second case, the tissue has partially left the slice and only half of the excited tissue experience the refocusing pulse, yielding intermediate signal intensity at the echo time. In the last case, the excited tissue has entirely left the slice before the refocusing pulse, resulting in no signal at the echo time. For fast spin echo techniques, where a train of refocusing pulses are applied for each excitation pulse, the blood suppression performance is given by the through‐plane flow at the effective echo time (TE_eff_) which is typically around the middle of the echo train (**b**). ES = echo spacing.

Through‐plane flow reduces spin echo blood signal, since only tissue that experience both excitation and refocusing radiofrequency (RF) pulses yield a spin echo. The slice‐selective nature of these pulses means that some blood flowing in the slice‐direction will only experience either the excitation or refocusing RF pulse, and thus not produce an echo at the echo time (TE). The amount of blood signal suppression due to through‐plane flow is related to the velocity (*v*) of the blood flow, the TE, and the slice thickness (*z*). Complete blood suppression is obtained for $v>\frac{2z}{\mathrm{TE}}$, as illustrated in Fig. 1a. This is the velocity at which blood travels through the imaging plane in less than TE/2, thus only experiencing either excitation or refocusing pulse. Based on this relationship, improved blood suppression can be achieved by reducing the slice thickness (at the expense of reduced SNR) and/or increasing the TE (leading to increased T_2_‐weighting). Improved through‐plane blood suppression can be achieved by synchronizing the acquisition with the cardiac cycle and timing the RF pulses to periods of high blood flow, such as arterial systole.

Spin echo images are often acquired using a train of refocusing pulses to accelerate the scan, called fast spin echo (FSE).^5^, ^6^, ^7^, ^8^ The single‐shot version of FSE is commonly used to rapidly obtain a complete 2D image with black‐blood contrast,^9^ illustrated in Fig. 1b. While the first echoes may contain some residual blood signal due to insufficient through‐plane flow, these encode peripheral *k*‐space when a linear phase encoding order is used. The effective contrast is instead given when the center of *k*‐space is acquired, much later in the echo train, which provides more time for through‐plane flow to occur. Similar to the single spin echo approach, increasing the effective echo time allows for better blood suppression but introduces T_2_‐weighting.

#### MOTION‐INDUCED INTRAVOXEL DEPHASING

The second mechanism that contributes to black‐blood contrast for spin echo is due to in‐plane flow, in particular in combination with a spatially changing magnetic field such as gradients or B_0_ inhomogeneities and some amount of transverse magnetization (M_xy_), which yields intravoxel dephasing. To understand this phenomenon, we will first define the relationship between spatial gradients, the motion of a spin, and its phase. Here we will consider the simple case of a spin with constant velocity motion *v*_*x*_ along direction *x*, which can be expressed as:

(1) $$x\left(t\right)=x_{0}+v_{x}t$$

where *x(t)* is the time‐varying position of the spin and *x*_*0*_ the position at *t* = 0. Higher‐order motion (such as acceleration, jerk, etc.) can be included in the motion equation by performing a Taylor expansion around *t* = 0 but will be ignored here.^10^ The time‐dependent phase *φ(t)* of the spin is given by:

(2) $$\varphi \left(t\right)=\gamma \int_{t_{0}}^{T}x\left(t\right)G\left(t\right)\mathrm{dt}$$

where G(t) is the time‐varying magnetic field gradient. Inserting Eq. 2 into Eq. 1 yields an expression for the phase that can be decomposed into phase terms that are dependent on position (*x*_*0*_) and velocity (*v*_*x*_):

(3) $$\varphi \left(t\right)=\gamma x_{0}\int_{t_{0}}^{T}G\left(t\right)\mathrm{dt}+\gamma v_{x}\int_{t_{0}}^{T}\mathrm{tG}\left(t\right)\mathrm{dt}$$

which after integration yields:

(4) $$\varphi \left(t\right)=\gamma x_{0}G\left(t\right)\left(T-t_{0}\right)+\gamma v_{x}\frac{1}{2}G\left(t\right)\left(T^{2}-t_{0}^{2}\right)$$

**FIGURE 2:**
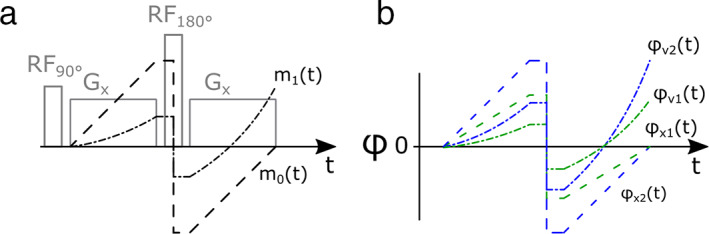
The evolution of the 0th and 1st gradient moment (m_0_(t) and m_1_(t), respectively) during a spin echo pulse sequence between the excitation (RF_90_) and echo at the end of the second gradient (G_x_) (**a**). Notably, m_1_(t) increase quadratically during the gradients and is nonzero at the echo time, unlike m_0_(t), which increase linearly. The corresponding phase for two spins with different position (x1 and x2) and velocities (v1 and v2) (**b**). The position‐dependent phase terms are nulled for both spins at the echo time while the velocity‐dependent phase is different, proportional to the differences in velocities and lead to dephasing if the spins are within the same voxel at the echo time.

From Eq. 4 we can observe that the position‐dependent phase term is linearly related to the duration of the gradient, while the velocity‐dependent phase term is quadratically related to the gradient duration. Note that each integration contains attributes of a gradient waveform (its strength and duration) and are typically referred to as the gradient moment of different order. Specifically, the product of the position of a spin and the 0th order gradient moment yields the position‐dependent phase, while the product of the 1st gradient moment and the velocity of a spin yields the velocity‐dependent phase. The relationship between the 0th and 1st order moments of gradients during a spin echo pulse sequence, and the resulting phase of two spins with different velocities are illustrated in Fig. 2.

**FIGURE 3:**
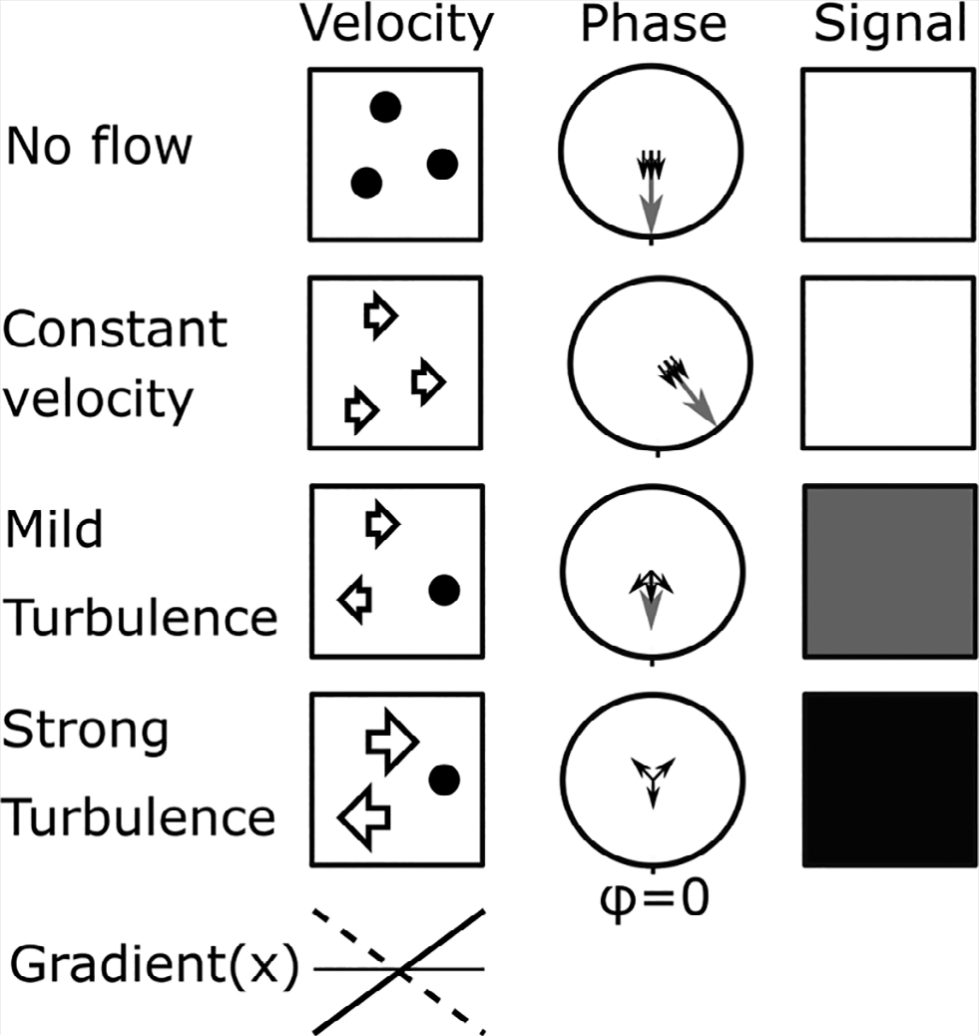
Dephasing of transverse magnetization due to velocity differences (turbulence) within a voxel. The left column illustrates three spins within a voxel for different flow conditions, no flow (top), constant velocity (second), mild turbulence (third), and strong turbulence (fourth), while there is an active bipolar gradient in the flow direction (x). The second column shows the resulting phase for the spins (individual spins in black, vector sum in gray) in the voxel where no or constant flow lead to the same phase for all spins, and subsequently maximum signal magnitude shown in the third column. Turbulent flow leads to a phase distribution across the voxel that results in a reduced magnitude, and complete signal suppression in the case of strong turbulence.

In MRI, particularly turbulent blood flow gives rise to intravoxel dephasing, where spins within a voxel have accrued different amounts of phase leading to reduced M_xy_ magnitude. However, it is important to note that if there is homogenous flow, the phase of all spins in a voxel changes by the same amount, and the voxel therefore maintains the same signal magnitude. This is the physical principle underpinning phase‐contrast MRI that has been extensively documented in the literature.^11^ The difference in magnetization magnitude and phase between blood experiencing no, constant, or variable flow and the resulting MRI signal are illustrated in Fig. 3. Black‐blood contrast in the context of dephasing of transverse magnetization is more akin to the signal loss caused by motion in diffusion‐weighted sequences. Gradients induce a spatially dependent phase which, coupled with motion during some mixing time, yields a distribution of phases for spins (isochromats) within a voxel and hence a reduced magnitude.^12^ However, here the context is not random Brownian motion but flowing blood with different velocities and spin history that mix, leading to black‐blood contrast. Like diffusion, increasing the moment of the gradients leads to stronger black‐blood contrast, either by higher gradient amplitude or increasing the mixing time. In practice, improving spin echo black‐blood contrast for in‐plane flow can be achieved by increasing the bandwidth (leading to stronger imaging gradients) or the echo time (leading to longer mixing time). It should be noted that both approaches reduce the SNR in general. Increasing the echo time, while adding to the black‐blood contrast caused by through‐plane flow, as outlined earlier, introduces T_2_ weighting.

**FIGURE 4:**
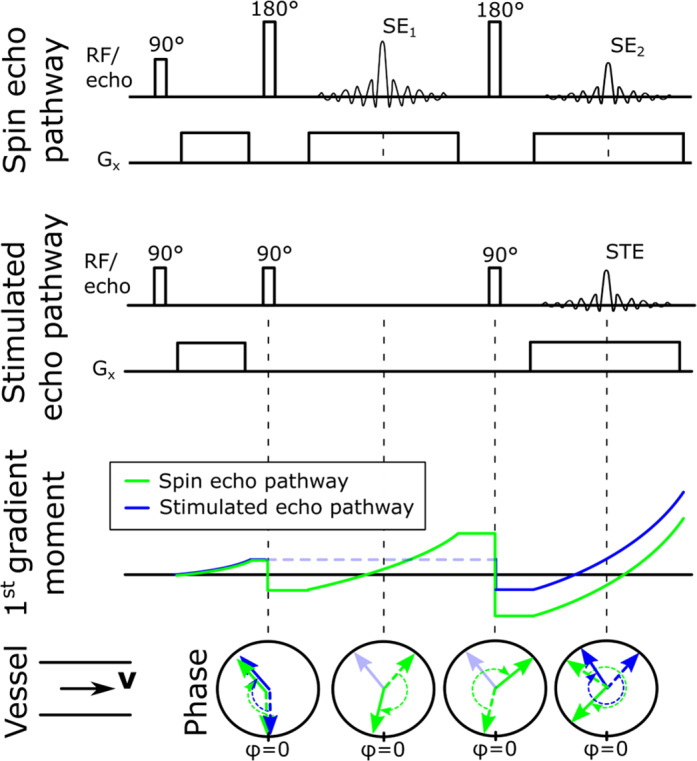
Dephasing of transverse magnetization during a fast spin echo sequence for spins moving through a vessel with constant velocity (v). If the actual refocusing pulses are lower than 180°, stimulated echo (STE) pathways will be created at each refocusing pulse. In this example, a portion of the M_xy_ magnetization at the second RF pulse are tipped back into the M_z_ direction and are not affected by the following readout gradient (G_x_), unlike the portion of M_xy_ following the spin echo pathway that remains in the transverse plane. The third RF pulse acts as an excitation pulse on a portion of the STE pathway and because the phase is stored, the following G_x_ yields a stimulated echo coinciding with the second echo of the spin echo pathway (SE_2_). However, differences in 1st gradient moment can lead to dephasing due to the motion‐induced phase difference between the SE_2_ and STE pathways. The illustration of phase shows the evolution of phase during the application of the RF pulses and gradients, where dashed straight arrows show the starting phase, the dashed curved arrows the change in phase and direction, and the solid straight arrows the final phase at each timepoint for the two spins following the spin echo (green arrows) and STE (blue arrows) pathway, respectively. Note the amount of spins that follow either spin or stimulated echo pathways will depend on the effective flip angle of the refocusing pulses.

So far in this section we have only considered the special case of a spin echo sequence where the measured signal is the result of one excitation and one refocusing pulse. In the case of FSE, where multiple refocusing pulses are performed for each excitation pulse the picture gets more complicated. The signal from spin echoes after subsequent refocusing pulses are subject to further intravoxel dephasing due to mixing of blood with different spin history within a voxel. Furthermore, if refocusing angles lower than 180° are applied, additional flow‐related signal loss—even for constant velocity flow—is caused by the phase disparities between the different echo pathways, including spin echoes and stimulated echoes, which lead to an echo formation with lower magnitude.^13^ In this scenario, the phase of the different echo pathways will depend on the time spent in the transverse plane (where phase is accrued proportional to the velocity and gradient strength) relative to the longitudinal plane (where phase is not accrued but stored), as illustrated in Fig. 4. Reducing the refocusing flip angles in 3D FSE sequences (variable flip angle FSE [VFA‐FSE]) is often implemented to allow extremely long echo trains with constant transverse magnetization for specific T_1_ and T_2_ combinations, has the benefit of lowering the specific absorption rate, and determines the contribution of spin echo and stimulated echoes in the echo train.^14^ For example, lowering the refocusing flip angles increases the relative contribution of stimulated echoes in later echoes and has the further effect of reducing the signal from flowing blood proportional to the refocusing angle.^15^ In general, the phase accrual in any flow direction is proportional to the moment of the applied gradients. The highest 1st gradient moment at any echo time tends to be in the readout direction, which is also why flow in the readout direction dephase faster relative to the phase and slice‐encoding directions.

In summary, spin echo techniques typically exhibit black‐blood contrast due to its high sensitivity to flow and motion. The black‐blood contrast is caused by a combination of one or more of the following effects that all reduce the blood signal: 1) *through‐plane flow*, leading to flowing blood not experiencing either the excitation or refocusing pulse, thus not creating a spin echo; 2) *motion‐induced intravoxel dephasing*, where phase is accrued in the transverse plane relative to the flow velocity and gradient strength, and mixing of blood with different phase accrual within a voxel due to flow turbulence lead to overall dephasing; and specifically for 3D VFA‐FSE; 3) *spin‐* and *stimulated‐echo pathway mixing* with refocusing angles less than 180°, causing flowing blood with any velocity to experience overall dephasing due to the differences in flow‐related phase created by the different spin and stimulated echo pathways.

So, what about gradient‐recalled echo (GRE) pulse sequences? Unlike spin echo techniques, GRE typically receives a signal boost from inflowing blood proportional to its velocity (the so‐called time‐of‐flight contrast mechanism), often massively outweighing any signal loss due to intravoxel dephasing (which is also minimized by using very short echo times) except for areas of highly turbulent flow. This explains why GRE is so popular for angiographic techniques, where bright‐blood contrast is desired. The GRE signal enhancement from inflowing blood is due to the conversion of blood with full longitudinal magnetization to a gradient‐echo with a single RF pulse, coupled with a short echo time on the order of a few milliseconds. However, introducing strong bipolar gradients with high 1st gradient moment prior to the GRE readout can enable black‐blood contrast,^16^ although it increases the shortest achievable echo time and T_2_* weighting. More commonly, magnetization preparation techniques, so‐called prepulses, can be used to combine the advantages of GRE (eg, motion robustness, rapid data acquisition, low specific absorption rate) with black‐blood contrast. In the following section we will review some prepulse techniques that can be used to yield black‐blood contrast with an arbitrary readout.

### Magnetization Preparation Pulses

Black‐blood prepulses typically either exploit the blood flow to achieve suppression, ie, *flow‐dependent* techniques, or rely on T_1_ and/or T_2_ differences between blood and vessel wall, ie, *flow‐independent* techniques, or a combination of the two.

#### FLOW‐DEPENDENT TECHNIQUES

The simplest flow‐dependent prepulse technique involves applying a slab‐selective saturation pulse (SAT) to blood flowing into the field‐of‐view (FOV).^17^ A time delay between the prepulse and imaging is required to allow the saturated blood to replace unsaturated blood in the FOV. However, the signal cannot be completely suppressed due to T_1_ recovery of the saturated blood, although the blood signal recovers slowly due to its long T_1_. Furthermore, the technique is primarily suited for FOVs oriented perpendicular to the flow direction to maximize the inflow of saturated blood. This limits its applicability in territories with complex tortuous vasculature or in the heart.

**FIGURE 5:**
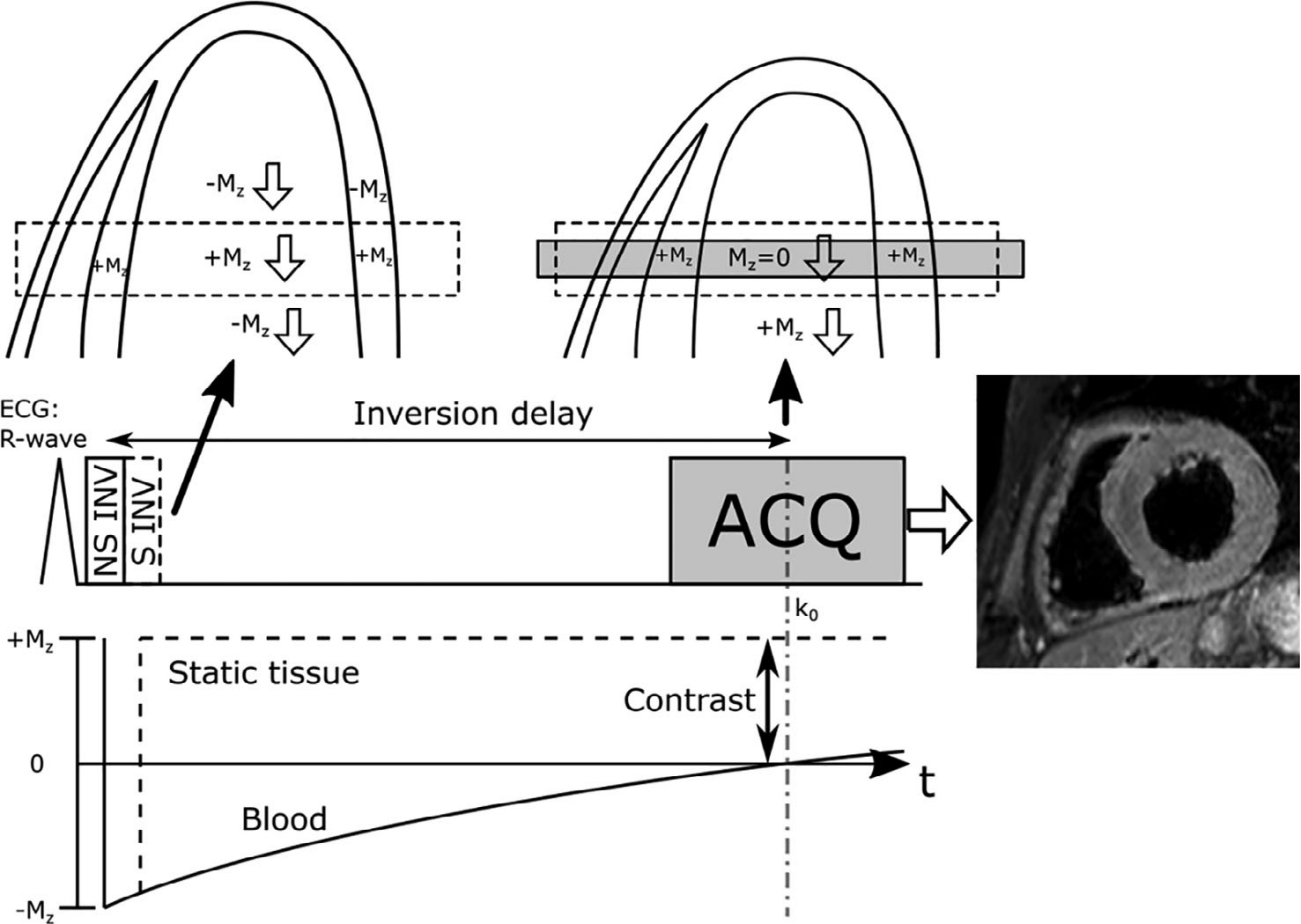
Sequence diagram for ECG‐triggered double‐inversion recovery in short axis view of the heart. A nonselective inversion pulse (NS INV) is applied after the R‐wave which inverts the longitudinal magnetization (M_z_) globally. This is immediately followed by a selective inversion pulse (S INV) which reinverts the area of the imaging slice (with some margin in the slice direction). The inversion delay is timed such that the inverted blood reaches the M_z_ zero‐crossing during the acquisition of the center of *k*‐space (k_0_), during which time it should also have replaced the reinverted blood that flows out of the imaging plane. This effectively nulls signal from blood flowing into the slice, while static tissue experiences both inversion and reinversion pulses and have full M_z_.

A widely used alternative to the saturation prepulse is the double‐inversion recovery (DIR) technique,^18^ illustrated in Fig. 5. Here, blood signal is “nulled” by first applying a nonselective 180° pulse that inverts the longitudinal magnetization (M_z_) with a delay such that the blood M_z_ reaches the zero‐crossing at the time of data acquisition. Although this in itself does not yield a high contrast due to the similar T_1_ of blood and vessel wall (which is almost nulled), a second slice‐selective reinversion pulse can be performed immediately after the nonselective pulse to restore the M_z_ within the FOV. The second inversion pulse restores the M_z_ of all tissue within the FOV, including the blood M_z_. However, during the time delay to null the inverted blood signal (on the order of several 100 msec), the inverted blood outside the FOV will have replaced the restored blood inside the FOV due to through‐plane blood flow. With sufficient through‐plane blood flow the DIR technique yields very high contrast between blood, vessel wall, and myocardium. However, the flow‐sensitivity of this technique is proportional to the slice thickness and better blood suppression performance is achieved for thin‐slice data acquisition. As a consequence, DIR is primarily only compatible with 2D coverage. To maximize blood suppression, the DIR prepulse is typically performed in early systole, immediately after R‐wave detection, while data acquisition is performed in diastole, which results in significant blood flow during the inversion delay period that covers the systolic period of high blood flow. Nevertheless, retrograde flow may cancel any forward through‐plane flow that can lead to suboptimal blood suppression, potentially mimicking or obscuring pathology. The DIR approach can be extended to incorporate fat suppression by adding a selective inversion pulse close to the data acquisition to null fat signal, so‐called short tau inversion recovery (STIR).^19^ A more advanced approach, called quadruple inversion recovery, incorporates a second DIR module to allow nulling of tissues within a high range of T_1_, including blood before and after contrast administration.^20^

Both SAT and DIR techniques rely heavily on inflowing magnetization‐prepared blood to achieve black‐blood contrast, and as such are primarily limited to 2D imaging to achieve sufficient inflow. To address this limitation, a prepulse exploiting the motion sensitivity of the spin echo sequence to intravoxel dephasing has been proposed, called motion‐sensitized driven equilibrium (MSDE).^21^, ^22^, ^23^, ^24^ The MSDE prepulse consists of a 90° pulse, tipping all M_z_ into the transverse plane, followed by refocusing pulses to counteract dephasing due to field inhomogeneities, followed by a 90° tip‐up pulse, similar to a T_2_‐prepulse to achieve T_2_ contrast.^25^ Strong gradients are applied before and after the refocusing pulse with a net zero 0th moment to refocus static spins but with nonzero higher‐order moment to induce intravoxel dephasing in voxels with blood flow. A technical challenge of MSDE is that strong gradients can introduce eddy currents that leads to signal loss. Furthermore, B_1_ inhomogeneities lead to incomplete refocusing, which again results in signal loss. Improved MSDE (iMSDE) techniques have been proposed that mitigate these problems, by introducing additional refocusing pulses.^26^

A further limitation of the MSDE approach is that it introduces T_2_‐weighting, due to the transverse relaxation of the magnetization between the 90° tip‐down and tip‐up pulses. Unfortunately, the relatively short T_2_ of static tissue (eg, vessel wall and myocardium) of ~50 msec, leads to a significant SNR penalty using the MSDE even if a short spacing is used between the 90° pulses. Delay alternating with nutation for tailored excitation (DANTE) is an alternative flow‐dependent prepulse technique that does not impose a T_2_‐dependent signal loss.^27^ In the DANTE prepulses, a train of ~150 nonselective, small flip angle RF pulses are applied with a short (1 msec) repetition time. Gradients are applied between the RF pulses, leading to a quadratically increasing phase for flowing spins in the gradient direction, while static spins have a linear phase accrual and maintain phase coherence. Effectively, this yields a dephasing of moving spins comparable to RF spoiling.^28^

#### FLOW‐INDEPENDENT TECHNIQUES

Flow‐dependent techniques exploit the differences in motion conditions between flowing blood and surrounding static tissue to generate contrast. However, in the case of stagnant blood or if the surrounding tissue moves due to, for example, cardiac or respiratory motion, black‐blood contrast can be significantly reduced. An alternative flow‐independent approach to blood suppression relies on differences in magnetic properties between blood and surrounding tissue to obtain black‐blood contrast.

Since T_1_ of blood (~1.6–1.8 seconds at 1.5T and 3T magnets) is typically only slightly longer than for static tissue (~1.0–1.2 second at respective field strengths), simply using an inversion pulse to yield T_1_‐dependent nulling of blood signal would also severely reduce the signal from static tissue. Therefore, an inversion recovery approach for blood suppression is typically not practical, with a few exceptions; for example, to visualize intraplaque hemorrhage of the arterial vessel wall^29^, ^30^ atrial ablation lesions in the acute phase^31^, ^32^ or the atrial wall.^33^ However, if T_1_‐shortening contrast agents are administered, such as for late gadolinium enhancement (LGE),^34^ inversion recovery with blood nulling may be a useful method to suppress blood signal while achieving sufficient signal from adjacent tissues.^35^ Techniques employing two inversion pulses, similar to quadruple inversion recovery, have been proposed to suppress blood signal in LGE.^36^, ^37^ In this approach the inversion pulses are timed to suppress signal from T_1_ over a certain threshold, including blood, while signal from scar (which has a low T_1_ due to the high gadolinium accumulation) remains high.

The T_2_ of oxygenated blood is ~5 times longer compared to that of the vessel wall, and this difference can be exploited to achieve flow‐independent blood suppression. Although T_2_‐weighting in itself cannot be used to suppress blood signal, a subtraction technique using a T_2_‐weighted and non‐T_2_‐weighted acquisition has been proposed, called interleaved T_2_ preparation (iT_2_prep).^38^ By acquiring one image with strong T_2_‐weighting using a T_2_ preparation module^25^ and one image without T_2_‐weighting, a subtraction between the images can effectively suppress blood signal because it is relatively unaffected by the T_2_ preparation, while signal from the vessel wall is strongly reduced. However, as a subtraction technique iT_2_prep is sensitive to motion between the acquisitions and also suffers from reduced SNR compared to nonsubtraction techniques.

The ability to provide excellent blood‐static tissue contrast using T_2_ preparation can be combined with the tissue nulling ability of T_1_ weighted inversion recovery to allow for flow‐independent black‐blood contrast, so called T_2_prep‐IR. Although this technique was originally proposed for bright‐blood angiography,^39^ it can be optimized to achieve black‐blood contrast by adjusting the inversion time and T_2_prep echo time.^40^ To mitigate the challenges of determining the precise inversion time to achieve blood nulling, this approach can be combined with phase sensitive inversion recovery (PSIR),^41^ commonly used to improve myocardial nulling in LGE.^42^ The T_2_prep‐PSIR approach involves acquiring a second interleaved image, without the T_2_prep‐IR pulses, which allows the determination of the polarity of the M_z_ magnetization during the acquisition of the magnetization‐prepared image. Although this doubles the scan time, it makes the acquisition insensitive to the precise inversion time for blood nulling and improves the vessel wall‐to‐blood contrast compared to T_2_prep‐IR. In the T_2_prep‐PSIR approach, the second image may be preceded by a T_2_prep module to enable the acquisition of both a black‐blood and bright‐blood dataset.^43^ A pulse sequence similar to T_2_prep‐PSIR (T_2_prep‐IR image acquired in one heartbeat and an image without prepulses in the following beat) can be used to yield black‐blood contrast through image subtraction.^44^ This is similar to the iT_2_prep approach, but without the need to calculate a subtraction factor to eliminate blood signal.

Contrast agents with high relaxivity of transverse magnetization such as ferumoxytol^45^ can be used to dramatically shorten T_2_ of the blood pool and allow blood suppression. In combination with a T_2_‐weighted FSE technique, this provided superior blood suppression compared to the T_2_‐weighted FSE technique alone.^46^ The effect of contrast agents on T_2_* is typically stronger than on T_2_ (in particular for superparamagnetic iron oxide agents such as ferumoxytol), and this can be exploited using spoiled gradient echo techniques to visualize both venous and arterial vasculature with black‐blood contrast.^47^

## Clinical Applications

Although the field of MRI contains a wide range of black‐blood techniques outlined in the previous section, in practice the optimal approach will depend on flow and motion conditions in the anatomy of interest, in addition to consideration of additional contrast weighting and spatial coverage. In the following section we will describe how black‐blood techniques have been implemented for specific anatomical regions and different pathologies, considering flow, motion, additional contrast weighting, and coverage.

### Myocardial Tissue Characterization

Characterizing myocardial tissue is extremely valuable in the clinical routine to differentiate between different cardiomyopathies and make accurate prognostic evaluations. This includes T_2_*‐weighted imaging that can indicate iron overload,^48^ T_2_‐weighted imaging that is related to edema,^49^ and T_1_‐weighted imaging that can be used to visualize myocardial fibrosis without^50^ and (more commonly) with gadolinium‐based contrast agents.^34^, ^51^ In this context, black‐blood contrast is useful to improve the delineation of the myocardium and reveal pathological signal changes that may otherwise be confounded by the bright‐blood signal.

#### T_2_ *‐ AND T_2_ ‐WEIGHTED IMAGING

The DIR prepulse has been implemented to achieve black‐blood contrast for quantitative T_2_*‐mapping of the myocardium^52^ and has been shown to improve reproducibility and have fewer artifacts compared to the bright‐blood alternative.^53^, ^54^ Myocardial T_2_‐weighted imaging is commonly combined with black‐blood contrast using the STIR prepulse.^19^ T_2_‐weighting is achieved using an FSE readout with an echo time of ~60 to 80 msec.^55^ However, the FSE technique is intrinsically sensitive to cardiac and respiratory motion that can cause motion‐related signal loss in the myocardium,^56^ although carefully timing the acquisition time to the cardiac rest period may mitigate cardiac motion artifacts.^57^ Furthermore, the STIR prepulse may fail to suppress slowly flowing or stagnant blood, causing edema‐mimicking artifacts.^58^ Finally, through‐plane cardiac motion between the initial inversion pulses and the image acquisition can cause the magnetization in the depicted myocardial tissue to be nulled, similar to inflowing blood. This may be compensated for by slice‐tracking to account for through‐plane cardiac motion,^59^ although it is nontrivial to accurately track and compensate for this complex motion. An alternative approach is to perform the selective inversion pulse in the preceding cardiac cycle, but the same cardiac phase as the imaging that minimizes through‐plane cardiac motion.^60^ Nevertheless, the susceptibility to artifacts of T_2_‐weighted STIR‐FSE has led to a questionable clinical usefulness,^61^ with bright‐blood T_2_‐weighted imaging using a flow‐ and motion‐insensitive sequence—T_2_prep with balanced steady‐state free precession readout^62^—showing better diagnostic accuracy.^63^, ^64^ Quantitative T_2_ mapping has been proposed as an approach to provide more objective detection of edema compared to T_2_‐weighted imaging. Again, spin echo‐based techniques have been proposed using DIR for enhanced black‐blood contrast.^65^ Although it may suffer from similar susceptibility to motion‐related artifacts as the T_2_‐weighted STIR‐FSE technique, it appears comparable to bright‐blood T_2_ mapping in terms of reproducibility.^66^

#### LATE GADOLINIUM ENHANCEMENT AND T_1_ MAPPING

**FIGURE 6:**
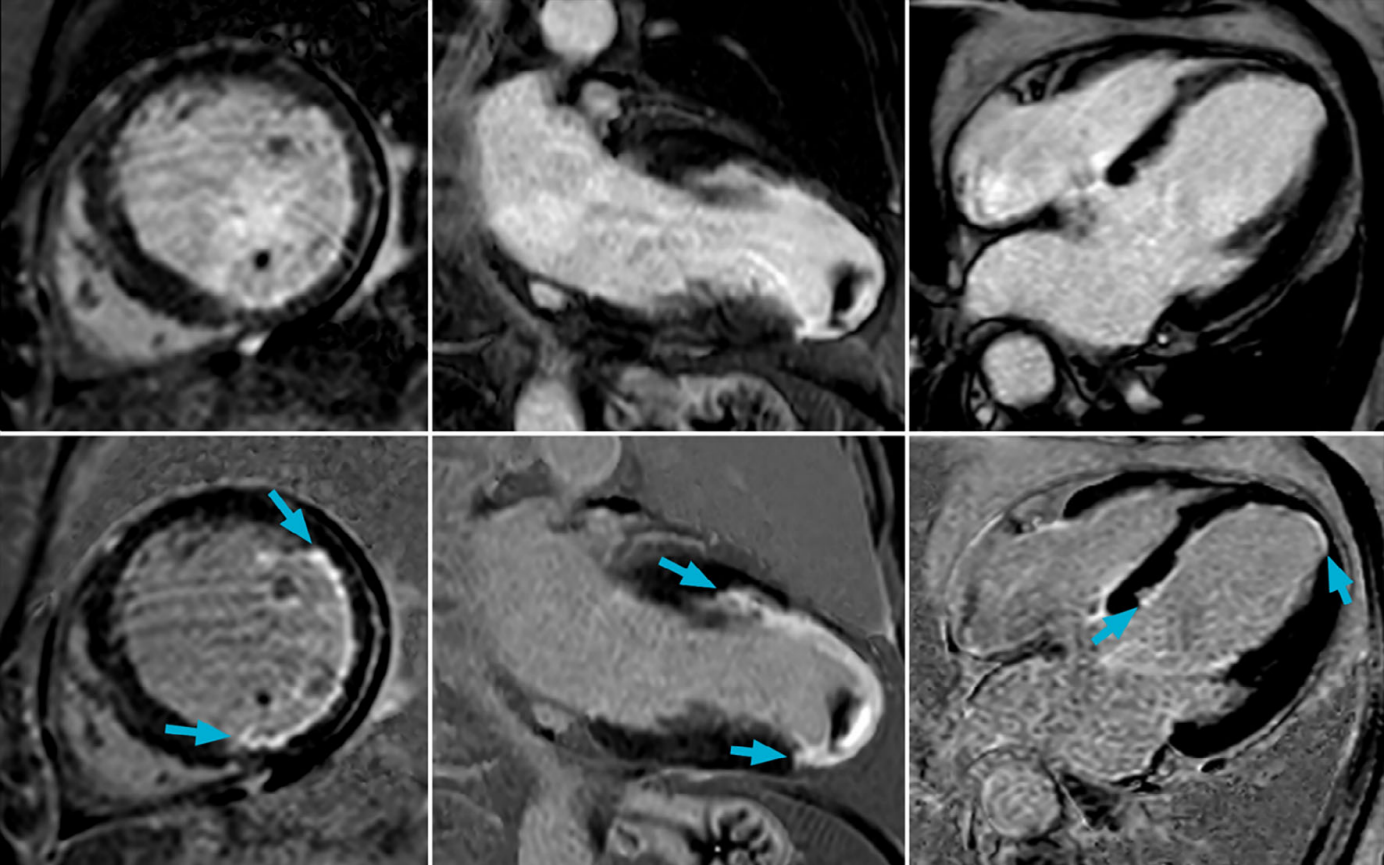
Conventional bright‐blood late gadolinium enhancement (LGE) (top row) and blood signal suppressed LGE (bottom row) in three patients with subendocardial and papillary muscle scar that can be clearly visualized using the dark blood technique. Images courtesy of Mr. Robert Holtackers, Maastricht University, Maastricht, The Netherlands.

Blood suppression for LGE is becoming increasingly popular because of its ability to enhance visualization of subendocardial scar,^67^, ^68^ as demonstrated in Fig. 6. Due to the administration of gadolinium‐based contrast agents with LGE, there is often a relatively wide disparity in both T_1_ and T_2_ between blood, healthy myocardium, and scar that allows several options for flow‐independent blood nulling using inversion and T_2_prep pulses. The simplest method involves adjusting the inversion pulse to null the blood signal rather than the myocardium.^69^ Combining several inversion pulses with carefully timed delays offers the possibility of suppressing a range of T_1_, including blood.^36^, ^37^ The addition of T_2_prep pulses before^70^ or after^71^, ^72^ the inversion pulse may also be used to suppress blood signal. Although in theory the T_2_prep module should be flow‐independent, as all RF pulses are nonselective, field inhomogeneities can cause flow‐induced dephasing.^73^ An alternative approach, which has a similar effect as T_2_prep, involves using a magnetization transfer (MT) prepulse in combination with inversion recovery.^74^ The MT pulse aims to selectively saturate magnetization of spins bound to macromolecules, which can be found in the myocardium, using a very high flip angle off‐resonance pulse.^75^ Finally, a combined T_1ρ_ and MT approach has been proposed, also incorporating inversion recovery, which lowers the requirement for high‐performance RF hardware compared to pure MT pulses.^76^ Instead of the T_2_prep or MT pulses, this approach uses a few net zero flip angle RF pulses, during which time T_2_ relaxation and MT occurs, prior to an inversion pulse to achieve blood nulling.

Black‐blood contrast is useful in T_1_ mapping for the same reason as LGE: to visualize subendocardial scar, but also to minimize partial volume effects that can affect T_1_ quantification. Compared to LGE, fewer techniques have been implemented, typically using MSDE prepulses for blood suppression.^77^, ^78^ The drawback of MSDE in this context is the introduction of T_2_‐dependent SNR loss, leading to a lower precision for T_1_ quantitation compared to bright‐blood T_1_ mapping.^78^ Furthermore, the MSDE prepulse is flow‐ and motion‐dependent and should ideally be performed in a cardiac motion‐free phase, which means there is less time for data acquisition during the cardiac rest period.

### Structural Heart Disease

**FIGURE 7:**
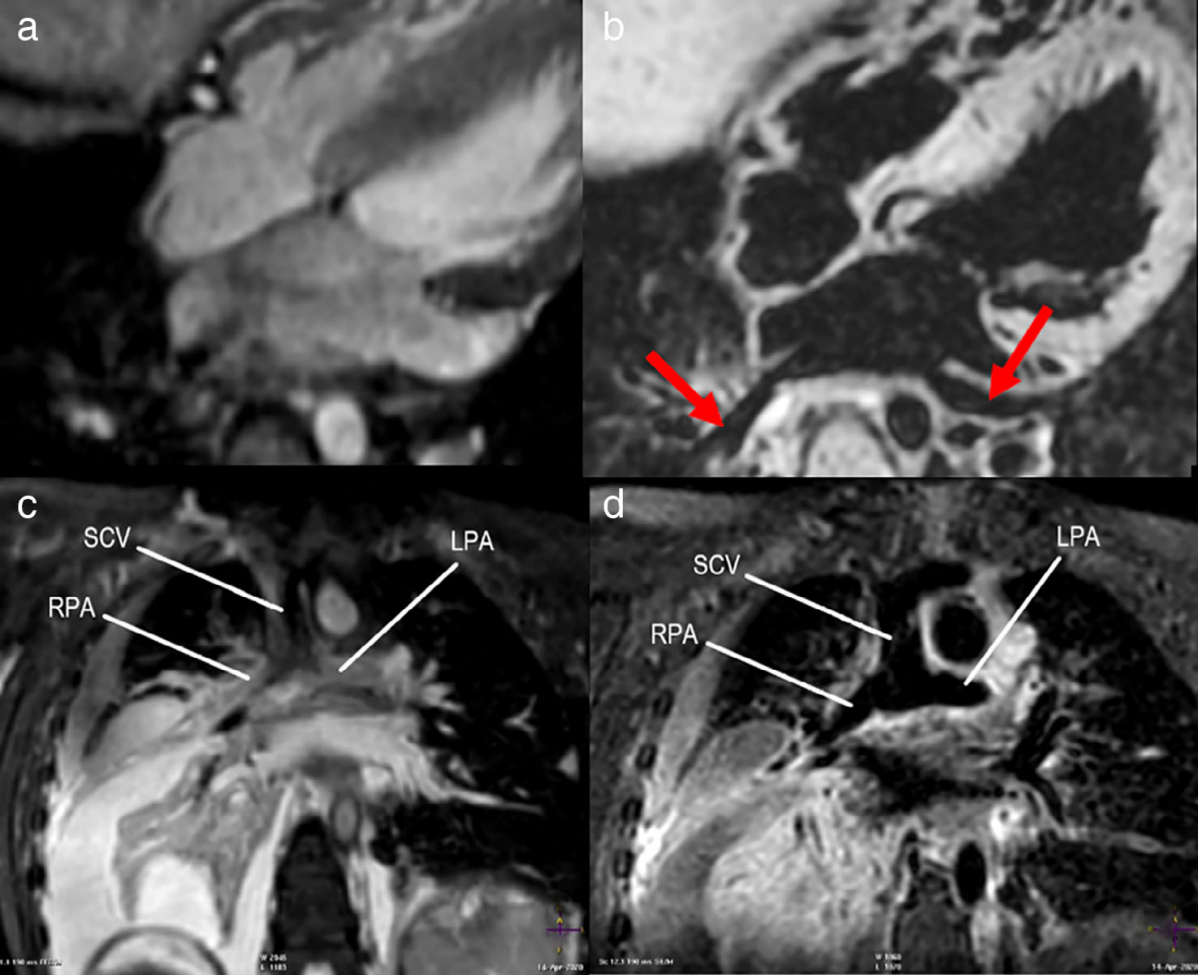
Images of pulmonary veins in a patient with congenital heart disease, where flow‐induced signal loss impede visualization using bright‐blood 3D bSSFP (**a**), while black‐blood 3D VFA‐FSE allow clearer depiction (red arrows) (**b**). Coronal view of superior caval vein (SCV), superior cavopulmonary anastomosis, and proximal branch pulmonary arteries in a 4‐year‐old female who underwent a superior cavopulmonary anastomosis (**c**,**d**). Three‐dimensional bSSFP demonstrates poor visualization of the SCV and proximal pulmonary arteries due to dephasing in the vessels (c), while 3D VFA‐FSE improves visualization of the SCV and branch pulmonary arteries (d). RPA = right pulmonary artery; LPA = left pulmonary artery.

Although structural heart disease covers a wide variety of pathologies, black‐blood techniques can often provide value in this setting. Particularly when imaging vessel walls, to avoid flow artifacts associated with bright‐blood techniques, and characterizing cardiac tumors, the pericardium or structures near devices such as stents. This has often been achieved using the 2D DIR technique, typically employing segmented *k*‐space FSE readout, which is more robust to the intravoxel dephasing caused by implanted devices compared to gradient echo techniques. ^79^, ^80^ Furthermore, 2D DIR FSE is compatible with T_1_‐ and T_2_‐weighting contrast, which is useful for tissue characterization.^81^ This is of particular importance in pericardial disease where T_1_‐weighted DIR allows visualization of pericardial morphology, while T_2_‐weighted DIR enables detection of pericardial fluid and edema.^82^ For morphological imaging of congenital structural heart disease, high‐resolution 3D coverage is typically preferred to capture the often irregular morphology that limits the applicability of DIR techniques. Recently, 3D VFA‐FSE was implemented for cardiovascular MRI and appears to improve depiction of the pulmonary veins compared to bright‐blood 3D balanced steady‐state free precession,^83^ an example of which is shown in Fig. 7a,b. In addition to the pulmonary veins, excellent visualization of other structures that may cause flow‐related dephasing using a bright‐blood technique in patients with congenital heart disease can be obtained using 3D VFA‐FSE, as shown in Fig. 7c,d. However, due to the motion‐sensitivity of this technique, it is important to ensure imaging is performed in a motion‐free cardiac phase.

**FIGURE 8:**
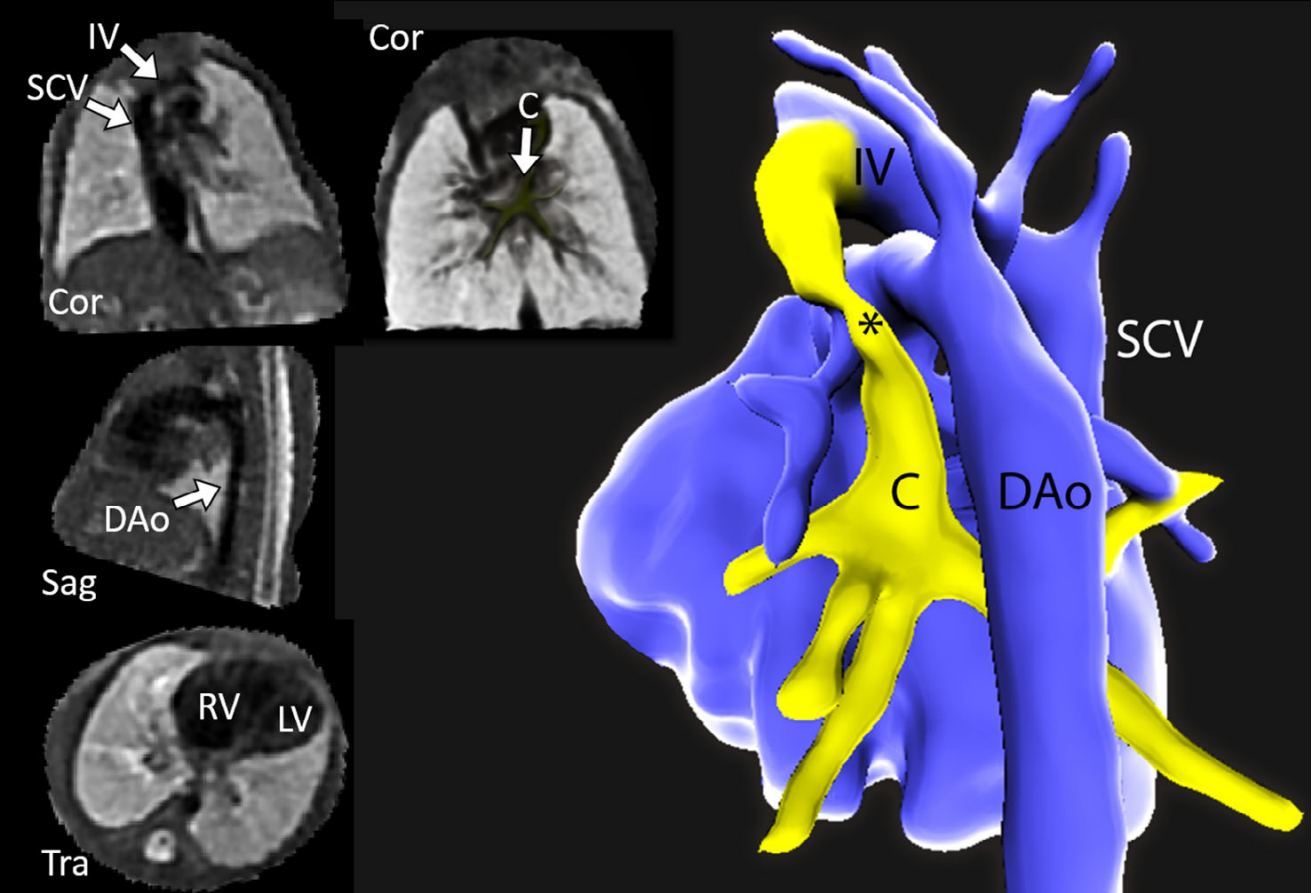
Black‐blood images from motion‐corrected volume (left), acquired from a fetus with hypoplastic left heart syndrome with total anomalous venous at 32 weeks gestation, displayed in coronal (Cor), sagittal (Sag), and transverse (Tra) planes. The 3D volume of fetal thorax was reconstructed retrospectively from multiple orthogonal input stacks of 2D images acquired using 2D FSE. A 3D segmentation of the fetal heart and vascular anatomy generated from this volume is shown on the right (posterior projection). * = ascending vein; IV = innominate vein; C = pulmonary venous confluence; DAo = descending aorta; SCV = superior caval vein; LV = left ventricle; RV = right ventricle. Images courtesy of Dr. David Lloyd, King's College London, London, UK.

Since the advent of fetal MRI, black‐blood techniques have played an important role to visualize structural heart disease.^84^ In particular, the single‐shot 2D FSE technique has been extensively used to this end due to its ability to obtain a relatively high spatial and temporal resolution, crucial requirements in the face of random and unpredictable fetal motion. The inability to employ electrocardiogram (ECG) triggering for MRI of the fetal heart, which is typically used for cardiac motion compensation, means that real‐time imaging is the only practical option. This limits the acquisition to 2D slices, where the black‐blood contrast is achieved by using thin slices (~2 mm) with echo times of 50 to 100 msec. Recent postprocessing techniques have allowed reconstructing volumetric cardiac images from multiple overlapping and orthogonal 2D slices,^85^, ^86^ as shown in Fig. 8.

### Vessel Wall Imaging

Black‐blood vessel wall imaging is a powerful technique to image various pathological conditions. The technique can be used to detect and characterize atherosclerotic plaques, aneurysms, dissections, and vasculitis in large and medium‐sized arteries. Challenges of vessel wall imaging are particularly related to the requirement for high‐resolution imaging, preferably on the order of submillimeter voxel size, in conjunction with the desire for multicontrast protocols to characterize pathology.

#### ATHEROSCLEROSIS

Atherosclerosis is a systemic disease characterized by fibrofatty intimal plaques with and without calcifications that may exhibit intraplaque hemorrhage. Due to its exquisite soft‐tissue contrast, MRI can distinguish these plaque components in large and medium‐sized arteries when using high spatial resolution black‐blood pulse sequences. Arguably, the most substantive and well‐validated work has been performed in the carotid arteries that are a prime target for vessel wall imaging because they are superficially located and provide a high signal using targeted surface coils.^87^ Due to the immobile nature of the carotid vessels, flow‐dependent black‐blood techniques can be readily used without risking signal loss due to motion. Flow‐dependent techniques were initially proposed using 2D DIR^17^ and 2D quadruple inversion recovery.^20^ More recently, MSDE^24^, ^26^ and DANTE^88^ prepulse techniques have been implemented that can be readily combined with high‐resolution 3D coverage. 3D VFA‐FSE has also been proposed, exploiting the intrinsic flow sensitivity of this technique for black‐blood contrast.^89^, ^90^ A recent technique permits 3D acquisition and simultaneous depiction of the vessel wall and the vascular lumen using a combined bright‐ and black‐blood imaging sequence.^91^ Modern black‐blood MRI techniques of the carotid bifurcation are highly accurate for identifying carotid atherosclerosis, including intraplaque hemorrhage^92^, ^93^ and patients at increased risk for stroke.^94^, ^95^, ^96^

**FIGURE 9:**
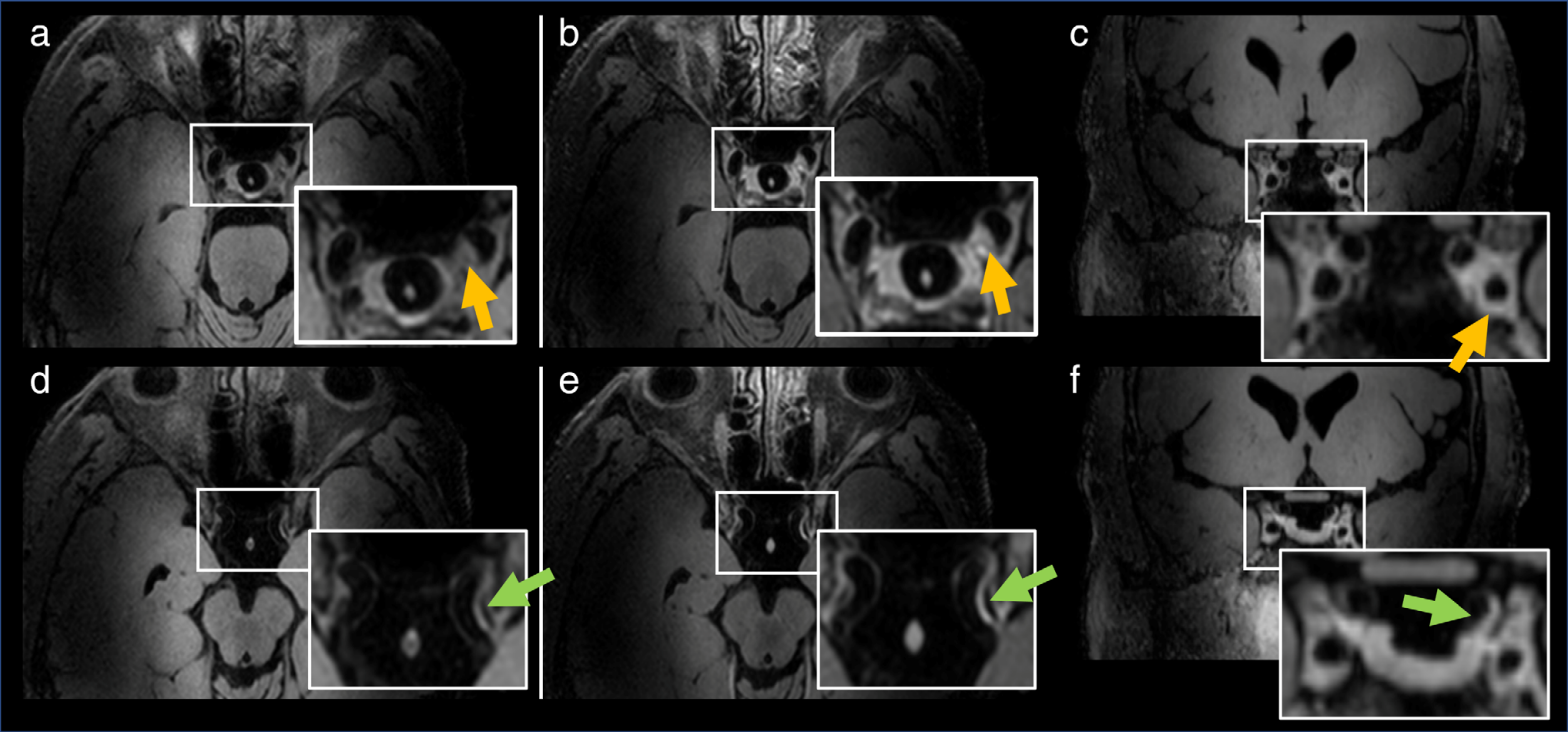
Forty‐seven‐year old male who presented with left‐sided transient ischemic attack. Pre‐ (**a**) and postcontrast (**b**) transverse source images of 3D VFA‐FSE show eccentric, enhancing atherosclerotic plaque in the carotid siphon (orange arrows). A second, smaller eccentric plaque further distally in the left intracranial carotid artery is seen in figure (**d**,**e**) highlighted by green arrows. In (**c f**) the corresponding postcontrast coronal reformations are shown. Images were acquired at 7.0T.

Although imaging the vessel wall of medium‐sized arteries is possible at a field strength of 1.5T,^97^, ^98^, ^99^ use of higher field strengths such as 3.0T and 7.0T allow 3D isotropic submillimeter spatial resolution acquisitions while maintaining sufficient SNR and has been shown to significantly improve coronary artery vessel wall image quality,^100^, ^101^ depiction of atherosclerosis in small intracranial arteries,^102^, ^103^ (Fig. 9) and even plaque rupture in patients with acute myocardial infarction.^104^ However, coronary vessel wall imaging is particularly challenging due to the presence of cardiac and respiratory motion that has stymied clinical uptake.^105^, ^106^ Larger patient studies—all using 2D DIR FSE—have reported analyzable vessel wall images in only 57–67% of patients.^107^, ^108^, ^109^ To mitigate against motion artifacts, advanced respiratory motion correction techniques have been implemented for 2D DIR^110^ and 3D iT_2_prep.^111^ Furthermore, patient‐specific triggering to the cardiac rest period tailored to the individual coronary arteries can reduce the influence of cardiac motion.^112^ Other vascular beds susceptible to atherosclerosis that are more stationary, including the abdominal aorta or the femoral arteries, have seen a wider use of volumetric black‐blood techniques using MSDE,^113^, ^114^ DANTE, 3D VFA‐FSE,^115^, ^116^, ^117^ or a combination of these.^118^, ^119^

#### ANEURYSMAL DISEASE

**FIGURE 10:**
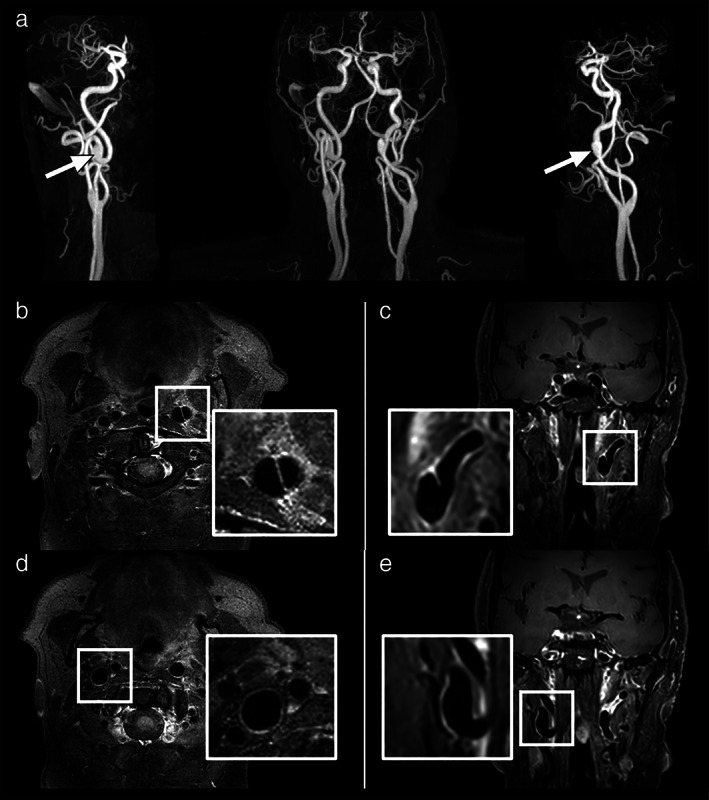
Sixty‐three‐year old male patient with bilateral carotid artery aneurysms. Maximum intensity projection of contrast‐enhanced MR angiography shows cervical and cranial vasculature in the coronal plane (**a**, center), and in double oblique projections of the right (a, left panel) and left (a, right panel) carotid arteries. White arrows denote the aneurysms. Contrast‐enhanced black‐blood 3D VFA‐FSE transverse source image (**b**,**d**) and coronal reformation (**c**,**e**) of the right and left carotid arteries show the enlarged vascular lumen and thin, heterogeneously enhancing vessel wall (b,d). Note excellent black‐blood contrast despite the presence of contrast agent.

Black‐blood imaging is also highly valuable for imaging of aneurysmal disease (Fig. 10) because it enables comprehensive depiction of the vascular lumen, the vessel wall, as well as any wall thrombus. Aneurysm vessel wall characteristics have been shown to be correlated with the presence of white matter lesions in the brain^120^ and clinical symptoms in the vertebrobasilar circulation.^121^ Furthermore, it is possible to obtain a rough estimate of thrombus age,^122^ to assess vascular and thrombotic remodeling over time, including growth rate of abdominal aortic aneurysms,^123^, ^124^ which can aid in clinical decision‐making regarding the need for endovascular or surgical interventions.

#### DISSECTION

Black‐blood vessel wall imaging has also been explored in the context of arterial dissection, primarily in the cervical and intracranial vasculature and using the 3D VFA‐FSE technique. The presence of luminal stenosis, aneurysmal dilatation, intramural high signal, and intimal flap/double lumen of the vertebral and basilar arteries were depicted successfully by Natori et al using black‐blood imaging at 1.5T.^125^ Arai et al performed high spatial resolution MR vessel wall imaging in patients with proven vertebrobasilar artery dissection and found vessel walls to be enhanced at the dissection sites in all patients.^126^ Zhu et al reported that 3D high spatial resolution MR vessel wall imaging could detect direct signs of dissection more frequently than catheter angiography, and that this aided in accurate differentiation between dissecting aneurysm and segmental ectasia.^127^

#### VASCULITIS

Vasculitis can affect arteries of any size and in any organ and clinical symptoms are primarily determined by the location and severity of arterial inflammation and resulting narrowing and hypoperfusion. Black‐blood MRI has long played an important role in detection and quantification of vascular wall inflammation in large and medium‐sized arteries. High spatial resolution 3D VFA‐FSE techniques at 3T are presently the most widely used and have been shown to be capable of detecting thoracic aortic and arch vessel vasculitis,^128^ abdominal aortic vasculitis,^129^ but also biopsy‐proven vasculitis in smaller branch vessels such as the superficial temporal and ophthalmic arteries and intracranial branches of the carotid artery.^130^ An important strength of MRI is that concomitant muscle involvement can be detected using the same acquisition.^131^ In combination with appropriate motion correction strategies, this approach can also be used for imaging the sequelae of coronary vasculitis in, eg, Kawasaki disease.^132^

To conclude this section on clinical applications of black‐blood MRI, we have listed key studies that have demonstrated the clinical value of black‐blood contrast for different cardiovascular applications in Table 1.

**TABLE 1:** Key Studies Demonstrating the Clinical Utility of Black‐Blood MRI

Study	Technique	Application	Clinical value
Smith et al^53^	2D DIR GRE: Two‐dimensional double‐inversion recovery gradient recalled echo	Myocardial T2* mapping	Fewer artifacts and better reproducibility vs. bright‐blood T2* mapping
O h‐Ici et al^133^	2D DIR FSE: Two‐dimensional double‐inversion recovery fast spin echo	Myocardial T2w imaging	Detection of acute myocardial infarction and distinguishing acute from chronic myocardial infarction.
Holtackers et al^69^	2D IR: Two‐dimensional inversion recovery	Myocardial LGE: Late gadolinium enhancement	Improved detection of subendocardial scar vs. bright‐blood LGE
Henningsson et al^83^	3D VFA‐FSE: Three‐dimensional variable flip angle fast spin echo	Structural heart disease	Improved pulmonary vein visualization compared to bright‐blood bSSFP: Balanced steady‐state free precession
Lloyd et al^85^	M2D single‐shot FSE: Multiple two‐dimensional fast spin echo	Structural heart disease (prenatal)	Accurate visualization of the fetal cardiovascular system
Wang et al^134^	3D VFA‐FSE	Carotid atherosclerosis	Excellent agreement with clinical gold standard, digital subtraction angiography (DSA)
Tian et al^135^	3D VFA‐FSE	Intracranial atherosclerosis	Good agreement with DSA and improved diagnostic performance compared to bright‐blood time‐of‐flight angiography
Zhu et al^127^	3D VFA‐FSE	Intracranial dissection	Superior ability to detect dissection vs. DSA
Maurus et al^129^	Contrast‐enhanced 3D VFA‐FSE	Large vessel vasculitis	Accurate depiction of vessel wall and inflammation
Xie et al^136^	3D DANTE VFA‐FSE: Thee‐dimensional delay alternating with nutation for tailored excitation fast spin echo	DVT: Deep vein thrombosis	Good agreement with contrast‐enhanced venography

bSSFP: balanced steady‐state free precession.

## Emerging Techniques and Applications

**FIGURE 11:**
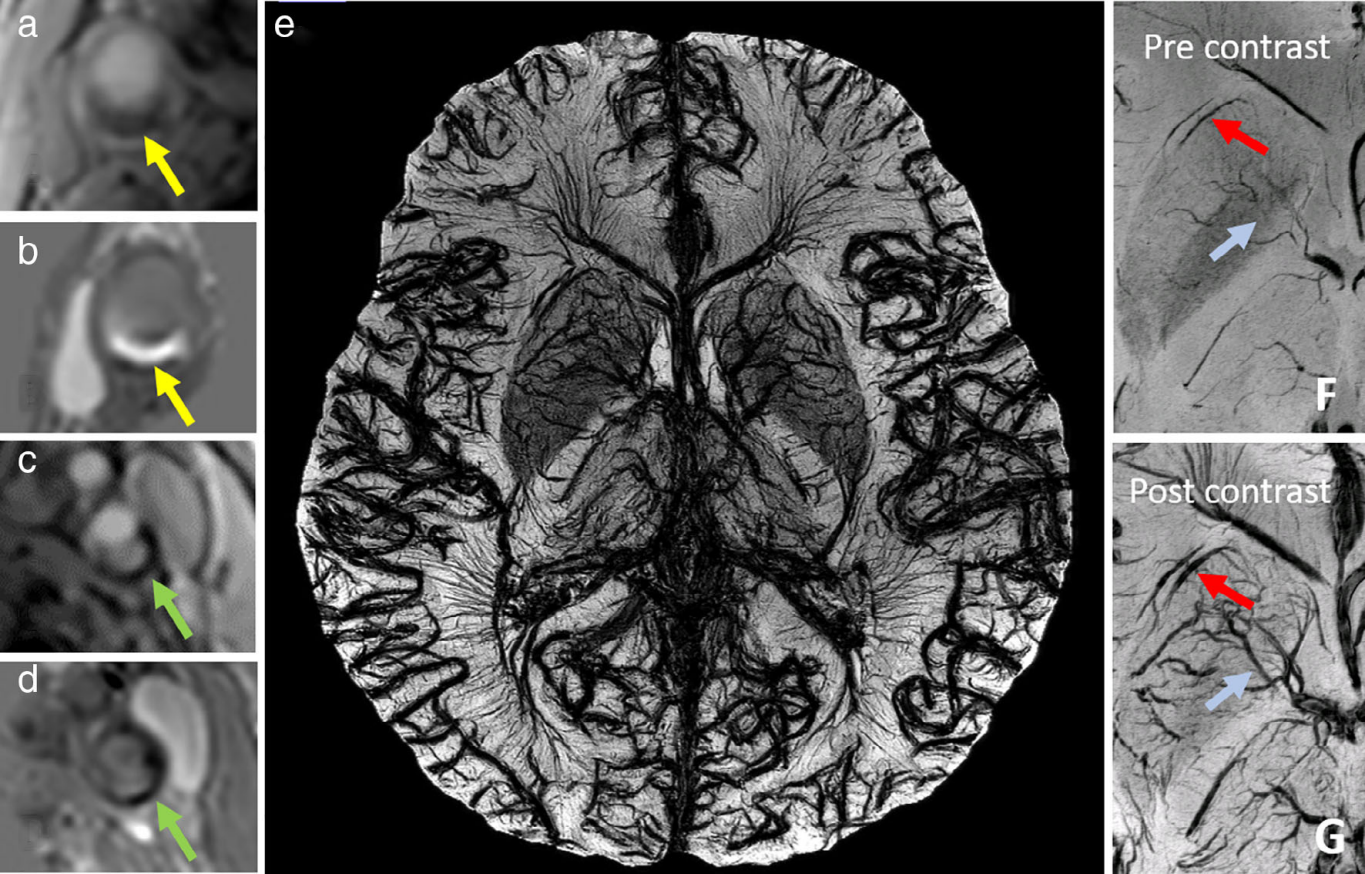
Susceptibility‐weighted imaging (SWI) from four subjects (**a**,**b**), (**c**,**d**), (**e**), and (**f**,**g**). To characterize vessel wall components of the carotid arteries, the magnitude images (a,c) are used for anatomical orientation and indicate areas of altered susceptibility at the vessel walls (yellow and green arrows). The phase image of the first patient shows a positive susceptibility that is indicative of intraplaque hemorrhage (b, yellow arrow), while the second patient has a negative susceptibility that suggests calcification (d, green arrow). Ferumoxytol‐enhanced brain SWI enable angiography and venography in a single scan (e). Before ferumoxytol injection SWI images yield black‐blood contrast for deoxygenated venous blood that has a relatively high susceptibility compared to surrounding tissue (red arrow) unlike arterial blood (light blue arrow) (f). Administration of ferumoxytol increases susceptibility in both venous and arterial blood that enables black‐blood venograms and angiograms (g). Images courtesy of Drs. Chaoyue Wang and Qi Yang, Capital Medical University, Beijing, China, and Dr. Mark Haacke, Wayne State University, Detroit, Michigan, USA.

A recent approach to visualize the vessel wall and characterize plaque, particularly calcification or intraplaque hemorrhage, is based on the susceptibility‐weighted imaging (SWI) technique.^137^ This contrast mechanism exploits differences in susceptibility between blood, calcified vessel wall, and/or hemorrhagic plaque. As a result of susceptibility differences, the resonance frequency is slightly different at the interface of these tissues, which appear as variations in the signal phase in a spoiled gradient echo sequence. However, the phase is affected by confounding factors such as blood flow and inhomogeneities of the static magnetic field. Therefore, first‐order gradient moment nulling is typically employed to eliminate the phase caused by constant velocity flow, while high‐pass filtering of the phase can be used to remove the slowly varying background phase changes caused by magnetic field inhomogeneities. The phase image itself may be used to visualize the vessel wall, provided any B_0_ inhomogeneities have been removed and phase aliasing unwrapped,^138^, ^139^ as shown in Fig. 11a–d. SWI also enables venograms with black‐blood contrast due to the paramagnetic properties of deoxygenated venous blood.^140^ This creates a susceptibility‐induced phase difference of venous blood relative to static tissue using GRE techniques. In practice, the signal phase is used to create a mask that is combined with the magnitude image resulting in black‐blood venograms,^141^ as shown in Fig. 11f. Using paramagnetic contrast agents such as ferumoxytol enables visualization of both arteries and veins using SWI, examples of which are shown in Fig. 11e,g.^47^, ^142^ In theory, SWI should be flow‐independent due to flow compensation gradients, as long as the flow is constant velocity. However, the need for low receiver bandwidth to obtain sufficient susceptibility contrast‐to‐noise results in very long repetition times, and the technique has primarily been applied in stationary tissue that does not require ECG‐triggering and where imaging can be performed continuously.

Another new technique to obtain black‐blood contrast (among other things) is so‐called magnetic resonance multitasking.^143^ This approach exploits the relatively slowly varying nature of the MR signal along certain dimensions (eg, T_1_ relaxation, cardiac and respiratory motion). Using a low‐rank tensor image model, highly undersampled images can be reconstructed at arbitrary timepoints on the inversion recovery curve for a certain cardiac and respiratory phase, such as at the zero‐crossing of the blood M_z_, yielding black‐blood contrast.^144^ A unique property of this technique is the ability to obtain time‐resolved black‐blood images that is typically limited to bright‐blood techniques.

Recently, black‐blood MRI has been applied to visualize DVT, a disease that primarily affects the lower extremities. Rapid and accurate diagnosis of thrombotic episodes is crucial in reducing the morbidity and potential mortality associated with arterial and venous thrombotic disorders by allowing early targeted therapeutic interventions. Unlike the arterial system, where the blood flow is highly pulsatile, with high flow during systole and low flow during diastole, venous flow is relatively constant throughout the cardiac cycle. Due to the predictable and constant flow conditions combined with the stationary nature of the lower extremities, flow‐dependent black‐blood techniques using 3D VFA‐FSE may be particularly well‐suited for DVT.^145^ Further suppression of slowly flowing blood has been achieved by combining 3D VFA‐FSE with the DANTE prepulse.^136^, ^146^ Black‐blood MRI allows accurate localization of the thrombus, which is challenging using conventional bright‐blood MRI where both venous blood and the thrombus yield a high signal, necessitating the use of contrast enhanced MR venography. An alternative to 3D VFA‐FSE for black‐blood MRI of DVT is the so‐called magnetic direct thrombus imaging (MRDTI).^147^ This technique allows to accurately assess the age and composition of thrombus, which is highly desirable given that anticoagulation and, in particular, fibrinolytic therapies are more effective in treating acute rather than chronic thrombosis.^148^ MRDTI is based on a T_1_‐weighted gradient echo pulse sequence where an inversion pulse is used for blood suppression. This technique can be used to distinguish fresh or recurrent deep venous thrombosis from persistent intravascular abnormalities in patients with a history of deep venous thrombosis and can be used safely as the sole test to decide whether or not to give anticoagulation therapy.^149^

## Artifacts in Black‐Blood MRI and Technical Challenges

Due to the wide range of black‐blood techniques, there are also a multitude of different types of associated artifacts. However, the most prevalent artifact in black‐blood MRI is the residual blood signal due to insufficient blood suppression. This is particularly problematic in cases where residual blood signal can mimic or obscure pathology, such as in T_2_‐weighted myocardial STIR, where stagnant blood near the pericardial border may appear as edema or DIR vessel wall imaging where slow or retrograde blood flow could be interpreted as atherosclerotic plaque. Although these may be considered worst‐case scenarios, even in less severe cases residual blood signal can reduce the contrast‐to‐noise ratio and lower diagnostic confidence. As we have discussed in this review, black‐blood techniques may be broadly classified as either flow‐dependent or flow‐independent techniques, including the spin echo sequence that can be considered flow‐dependent. Residual blood signal artifacts typically appear in flow‐dependent techniques in the case of stagnant or slowly flowing blood. While in most cases it is possible to increase blood suppression sensitivity for flow‐dependent techniques, it is often at the expense of reducing the SNR or introducing T_2_‐weighting. Perhaps an even more problematic consequence of increasing sensitivity to slowly flowing blood is the risk of motion‐related signal loss of otherwise static tissue. This may include signal loss of the myocardium due to cardiac or respiratory motion, or from the vessel wall due to compliance during the arterial pulse pressure wave. Synchronization with the cardiac motion using, for example, ECG allows triggering the flow sensitization module (prepulse or data acquisition) to a specific cardiac phase that can reduce signal loss due to cardiac motion or pulse‐pressure‐induced wall motion at the expense of prolonging scan time and introducing susceptibility to heart rate variability.^150^

Flow‐independent techniques overcome many of the challenges of suppressing slowly flowing blood and motion‐induced signal loss of static tissue. For example, even if blood signal is insufficiently suppressed using some version of flow‐independent inversion‐recovery for blood nulling, PSIR can be employed to achieve maximal contrast and retrospective blood nulling. However, flow‐independent techniques have their own limitations and associated artifacts. Most flow‐independent techniques rely on T_1_ and/or T_2_ differences of blood and static tissue. However, the endogenous difference in T_1_ yields a relatively low contrast with a very low SNR of static tissue when blood is nulled. Furthermore, the much longer T_2_ of blood relative to static tissue means that this difference cannot be directly exploited for blood suppression. Contrast agents often enhance the T_1_ and T_2_ differences between blood and pathology that improves the applicability of flow‐independent techniques, although the T_1_ and T_2_ at any given time may vary (eg, due to contrast washout or heart‐rate changes) which may yield suboptimal contrast. The nominally flow‐independent T_2_ preparation module may in practice result in flow‐dependent blood signal modulation that can cause artifacts. Finally, because flow‐independent techniques are based on prepulses, they are often optimized to achieve blood suppression for a particular timepoint, when the center of *k*‐space is acquired, and are therefore less suited for long readout trains or non‐Cartesian trajectories where the center of *k*‐space is repeatedly sampled.

## Conclusion

With the increased adoption of high‐resolution, multicontrast MRI to characterize tissue near vasculature, including the vessel wall, the development of black‐blood contrast mechanisms has similarly received increasing attention in the last decade. Due to the susceptibility of black‐blood techniques to artifacts (eg, motion and flow) and the need to trade off blood suppression for other image parameters (eg, SNR, spatial coverage, and T_1_‐ or T_2_‐weighting), development of optimal black‐blood strategies for different vascular beds is very much an active research topic. However, the general trend in the field for black‐blood MRI of noncardiac vasculature appears to be converging on the 3D VFA‐FSE technique. The advantages of this approach are the ability to achieve T_1_‐ or T_2_‐weighting with high resolution and volumetric coverage in a relatively short scan time. By combining this approach with blood signal suppressing prepulses, most popularly using DANTE or MSDE, additional suppression of slowly flowing blood may be achieved. However, the flow‐dependent 3D VFA‐FSE technique requires the specific condition to be met of relatively high blood flow with little to no motion of the static tissue, which is difficult to achieve in the nearly continuously moving heart. This is why alternative approaches have been more successful for black‐blood imaging in the heart and great vessels. Despite its many limitations (small coverage, susceptibility to motion and flow artifacts) 2D DIR‐FSE remains the most common noncontrast technique clinically for black‐blood imaging of the heart. This is largely due to its short scan time, which typically eliminates respiratory motion artifacts, as it can be performed in a breath‐hold, while blood signal suppression is often adequate. Several noncontrast techniques have been proposed, both flow‐dependent and flow‐independent, to overcome the drawbacks of 2D DIR‐FSE, particularly to allow volumetric black‐blood imaging of the heart. However, the challenge of long scan time intrinsic to volumetric, high‐resolution MRI has so far hindered widespread application of these techniques, as it increases susceptibility to respiratory motion artifacts. In this context, the combination of volumetric black‐blood cardiac MRI with recent image acceleration techniques such as compressed sensing may prove particularly beneficial to facilitate clinical translation. Compared to noncontrast cardiac MRI, more headway has been made for contrast‐enhanced black‐blood cardiac MRI, with many recent technical and clinical studies demonstrating the added value of flow‐independent black‐blood LGE relative to the conventional bright‐blood alternative. Flow‐independent techniques ensure homogeneous blood signal suppression, and rely on T_1_ and T_2_ differences between static tissue and blood to suppress blood signal, differences that are exacerbated after contrast agent injection. Recommendations for black‐blood sequences for different anatomical areas are provided in Table 2.

**TABLE 2:** Black‐Blood Pulse Sequence Recommendations

Type	Anatomy	Prepulse	Resolution	Orientation	Contrast	Comments
3D VFA‐FSE	Intracranial	DANTE/MSDE^a^	0.6 mm iso	TRA/COR/SAG	T_1_w, T_2_w or PD	
	Carotids	DANTE/MSDE^a^	0.8–1.0 mm iso	TRA/COR	T_1_w, T_2_w or PD	
	Aorta, thoracic & abdominal	DANTE/MSDE^a^	1.2 mm iso	SAG (thor), COR (abd)	T_1_w, PD	ECG/PPU‐trig
	Periperal	DANTE/MSDE^a^	1.0–1.5 mm iso	COR	T_1_w, T_2_w or PD	ECG/PPU‐trig
2D FSE	Cardiac	DIR	1.5 × 1.5 × 6 mm	Oblique/perpendicular to vessel	T_1_w, T_2_w or PD	ECG‐trig & BH
2D GRE	Cardiac (CE)	IR	1.8 × 1.8 × 8 mm	Oblique	T*1*w	ECG‐trig & BH. TI null blood.

CE = contrast enhanced; TI = inversion time.

^a^ Prepulses optional.

In summary, the optimal black‐blood techniques are highly dependent on the specific flow and motion conditions of the imaged anatomy. Challenges also relate to the desire for volumetric coverage with high resolution, where the former can be a direct impediment to some flow‐dependent black‐blood techniques. Great strides have been made in recent years to translate 3D high‐resolution variable flip angle fast spin echo into clinical practice for many applications where motion conditions are benign (intra‐ and extracranial arteries and peripheral vasculature). However, robust black‐blood contrast for cardiac MRI remains an elusive goal, although promising techniques are in the early stages of clinical translation.
